# Entropy generation minimization of higher-order endothermic/exothermic chemical reaction with activation energy on MHD mixed convective flow over a stretching surface

**DOI:** 10.1038/s41598-022-22521-5

**Published:** 2022-10-21

**Authors:** B. K. Sharma, Rishu Gandhi, Nidhish K Mishra, Qasem M. Al-Mdallal

**Affiliations:** 1 Department of Mathematics, Birla Institute of Technology and Science Pilani, Pilani, Rajasthan India; 2 Department of Basic Science, College of Science and Theoretical Studies, Saudi Electronic University, Riyadh, 11673 Saudi Arabia; 3 Department of Mathematical Sciences, College of Science, UAE University, P.O. Box 17551, Al-Ain, United Arab Emirates

**Keywords:** Energy science and technology, Engineering, Mathematics and computing, Physics

## Abstract

The present investigation aims to analyze higher-order endothermic/exothermic chemical reactions with activation energy by considering thermophoresis and Brownian motion effects on MHD mixed convective flow across a vertical stretching surface. The influence of velocity slip, thermal slip, and concentration slip along with an inclined external magnetic field is also considered. The governing coupled non-linear partial differential equations are transformed into ordinary differential equations using similarity transformation. The resulting system of non-linear ODEs is solved by the Newton Raphson shooting technique using the RK-4 algorithm. The impact of various physical parameters discovered in the problem viz. endothermic/exothermic reaction variable, thermophoresis parameter, activation energy parameter, Brownian motion parameter, chemical reaction parameter have been analyzed on velocity profile, temperature profile, and concentration profile. The effects of these parameters on skin-friction coefficient, Nusselt number, and Sherwood number are displayed in tabular form as well as surface plots. The impact of various physical parameters that appeared in the entropy generation is shown using surface and contour plots. The numerical findings are in good agreement with the previously published results. It is observed that an increment in thermophoresis and Brownian motion parameters results in a declination of entropy profiles, whereas an increment in Bejan number profiles is observed. A small region near the surface exhibits an inclination in concentration profiles with an increase in the order of the chemical reaction. In contrast, the opposite effect is analyzed near the boundary layer. Also, the contour and surface plots are displayed to portray real-world applications in industrial and technical processes and the physical depiction of flow characteristics that arise in the current study.

## Introduction

The mixed convection flows with simultaneous heat mass transfer involving Arrhenius activation energy with chemical reactions have been studied in recent years due to their vast applications. Most industrial end products’ quality is determined by cooling rates and chemical reactions, either the reaction rate or the type of chemical reaction. The present model includes activation energy, which most researchers have not included in earlier studies. Activation energy is widely considered while studying various physical phenomena, such as oil storage and engineering. A few theoretical publications on the role of activation energy in fluid dynamics are available. In 1889, Arrhenius made a groundbreaking attempt to present the concept of activation energy. The activation energy is the smallest amount of energy required by the reactants for a chemical reaction to occur. This phenomenon is used extensively in nuclear reactors, oil and water emulsion mechanics, and food processing. Menzinger and Wolfgang^1^ explained the detailed meaning of Arrhenius energy. Bestman^2^ was the first to develop and study this phenomenon in boundary layer transport. Makinde et al.^3^ investigated unsteady natural convection flow numerically under the impact of nth-order chemical reaction and activation energy. In the presence of thermal radiation, Maleque^4^ analyzed the effects of endothermic/exothermic chemical reactions having Arrhenius activation energy on MHD free convection flow. Shafique et al.^5^ used a numerical technique to quantitatively report a rotating viscoelastic flow with activation energy. Tripathi et al.^6,7^ discussed the influence of chemical reaction on blood flow considering variable viscosity model. Dhalmini et al.^8^ addressed the entropy generation and activation energy in viscous nanofluids containing higher-order chemically reactive species. Ullah^9^ investigated the activation energy associated with exothermic/endothermic reactions on magnetized nanomaterials flowing through a Darcy–Forchheimer porous medium. Dawar et al.^10^ studied mixed convective MHD flow of magnetite ferroparticles (Fe$_3$O$_4$) with blood as a base fluid past a non-isothermal vertical flat plate. Dawar et al.^11^ performed a mixed convective MHD flow of a water-based Al$_2$O$_3$ nanofluid towards the stagnation region of an angularly revolving sphere.

In the intervening years, a considerable amount of attention has been received by combining heat and mass transfer problems with chemical reactions. The temperature differences lead to heat transfer. Therefore an enormous variety of heat transfer equipment was created to address these differences, such as boilers, condensers, radiators, furnaces, refrigerators, solar collectors, compact heat exchangers, and many more. The impact of a drop of dye in water is an example of mass transfer. Mass transport has many industrial applications. Air and water pollution processes are also diffusion controlled. Heat and mass transport co-occur in various processes, such as flow in a desert cooler, evaporation from the top of any water body, and energy transfer in a wet cooling tower. Kandasamy et al.^12^ researched the impact of heat and mass transportation with the thermal stratification effects on MHD flow through a stretching surface. Sharma et al.^13^ discussed the transfer of mass and heat on 3D flow through porous medium. Rajeswari et al.^14^ studied the influence of suction on heat and mass transport through a porous vertical surface. Ahmad and Khan^15^ studied heat and mass transmission with viscous dissipation over a moving wedge. The influence of heat and mass transport on nanofluid flow over a stationary/moving vertical plate embedded in a porous media was investigated by Madhura et al.^16^. Sharma and Kumawat^17^ studied heat and mass transportation considering Ohmic heating and variable viscosity effects via a stretching surface. The heat transfer analysis of a water-based hybrid nanofluid, including ferrous and graphene oxide nanoparticles, over a flat plate was studied by Dawar et al.^18^ using magnetohydrodynamics.

MHD is important to cover the liquid’s mechanical properties in fluid dynamics and deals with the cooperation among the electrically conductive and electromagnetic fluids. The current may be generated whenever the conductive liquid particles move under the concurrent action of the electric and magnetic fields. The interaction with the magnetic field contributes to a body force on the liquid. MHD flow takes place in the sun as well as in the interior of the earth. Many new devices in the laboratory make full use of the MHD interaction, such as propulsion units and power generators, including interactions between fluid and electromagnetic fields, such as dynamics of electron beams and traveling wave tubes. As many critical engineering and industrial applications exist, combining mass and heat transport by MHD flow with chemical reaction inclusion has also received ample attention. Mansour et al.^19^ considered the presumptions of Soret and Dufour effects on the transportation of heat and mass through MHD free convective flow. Samad et al.^20^ explored heat and mass transport free convective flow with heat generation considering the magnetic field. Rajesh^21^ studied the impact on MHD free flow of thin gray fluid. Jafar et al.^22^ studied the transportation of heat and MHD flow over shrinking or stretching sheets. Under the presumption of varying permeability, Sharma et al.^23,24^ reported the chemical reaction’s influence on the micropolar fluid flow that replicates the microscopic effects owing to the local behavior and micro-motion of the liquid particles. Waqas et al.^25^ examined the micro-level impact of micropolar liquid on MHD flow through a non-linear sheet under the convective condition. Srinivasulu et al.^26^ investigates the effect of an aligned magnetic field with convective boundary conditions using numerical methods through a stretching surface. The impact of an induced magnetic field on the flow of Maxwell nanofluid toward a vertically permeable and stretchy sheet was explored by Walelign et al.^27^. Dawar et al.^28^ investigated the effect of an inclined magnetic field and inter-particle spacing on the two-dimensional flow of an electrically conducting water-based copper nanofluid over a stretching surface using a porous medium. Gandhi and Sharma^29^ studied MHD two-dimensional pulsatile blood flow through a vertical artery with irregular stenosis. A comparison of the magnetohydrodynamic flow of copper and copper oxide hybrid nanofluids based on water and kerosene oil across a bi-directional stretching surface was conducted by Dawar et al.^30^.

Thermophoresis is a temperature gradient-induced migration of suspended particles from higher to lower locations. Nuclear power plants, micro contamination, and aerosol collector are examples of their use. The suspended particles or water droplets in the air are known as aerosols. The thermophoretic force is the force that causes these aerosol particles to migrate due to a temperature gradient. Brownian motion is the ‘indecisive’ random movement of particles suspended in a fluid due to collisions with the fluid’s fast-moving molecules. Thermophoresis and Brownian motion are essential in heat and mass transmission in fluids. Hayat et al.^31^ examined MHD squeezing flow across a penetrable stretching surface in the presence of nanoparticles under the effect of Brownian movement. Sulochana et al.^32^ investigated the impact of stagnated point flow of a Carreau fluid past a shrinking/stretching plate with Brownian movement and MHD. Reddy et al.^33^ studied flow through a stretching geometry considering the effects of varying drag force and thermophoresis. Shah et al.^34^ studied thermophoresis’s variation on 3D nanofluid flow using a rotating system between parallel plates. Considering the non-Newtonian Prandtl fluid approach, Soomro et al.^35^ examined the effects of Brownian motion and thermophoresis via a vertical stretching surface on the MHD stagnation-point flow of nanofluid. The investigation of heat and mass transport in MHD Williamson nanofluid flow over a vertical Riga plate with nonlinear thermal radiation was carried out by Rooman et al.^36^. Dawar et al.^37^ addressed thermophoresis and Brownian motion effects considering Cu and CuO nanoparticles of different shapes.

The irreversible processes of viscous dissipation and Joule heating show how electrical and kinetic energy is transformed into thermal energy. Viscous dissipation is the work done via the fluid on nearby layers due to shear forces. In contrast, Joule heating is a mechanism in which conduction electrons transfer into the atoms of a conductor due to a collision procedure. Beg et al.^38^ investigated Joule heating effects on MHD Hartmann-Couette flow with Hall current. To introduce the outcome of viscous dissipation, Sahoo^39^ presented the second-grade MHD fluid flow past a transversally stretching surface. Many researchers^40–42^ discussed the influence of Joule heating with various additional conditions such as MHD flow, solar radiations, convective boundary conditions, and partial slip. Hsaio^43,44^ discussed the impact of viscous dissipation on coupled electrical MHD heat transfer and micropolar nanofluid flow. Gayatri et al.^45^ explored the Joule heating effects and viscous dissipation across a stretching sheet with varying thickness, utilizing slip parameters. Seethamahalskshmi et al.^46^ investigated the MHD mixed convective flow through a semi-infinite vertical plate under the influence of Joule heating and viscous dissipation using the two-term perturbation technique. Gandhi et al.^47^ analyzed the simultaneous effects of viscous dissipation and Joule heating through a stenosed artery considering the variable viscosity model.

Different forms of thermal systems are linked to the irreversibility process, which may be characterized using entropy generation, and are significant to viscous dissipation, magnetic fields, heat and mass transport, etc. Various studies employed the first law of thermodynamics to improve this irreversibility process, but the results were insufficient. Later, several researchers used the second law of thermodynamics to optimize these irreversibilities, concluding that the 2nd law is more efficient than the 1st. Rashidi et al.^48^ explored entropy generation in a rotating disc MHD fluid flow with variable properties. Dalir et al.^49^ used the Keller-box scheme to investigate the entropy formation for MHD heat and mass transfer flow of Jeffrey nanofluid across a stretched sheet. Baag et al.^50^ computed entropy generation by applying the second law of thermodynamics to MHD heat and mass transfer of an electrically conducting viscoelastic fluid past a stretching surface considering Darcy dissipation besides viscous and Joule dissipation. Bhatti et al.^51^ investigated entropy generation on the boundary layer flow with chemical reaction effects. Khan et al.^52^ performed entropy generation analysis in a mixed convective flow of nanomaterials with thermophoresis and Brownian movement considering the Buongiorno nanofluid model. Sohail et al.^53^ performed entropy generation computation for Casson fluid past a bi-directional stretched surface with heat and mass conveyance having a variable thermal conductivity. Hayat et al.^54^ studied irreversibility in the Darcy–Forchheimer flow of nanofluid by a curved stretching sheet with a spiral shape. Sharma et al.^55^ performed entropy analysis through a multi-stenosed artery in the prsence of hybrid nanoparticles (Au-Al$_2$O$_3$/Blood). Gandhi et al.^56^ conducted an entropy analysis of the MHD blood flow of hybrid nanoparticles of various shapes through an irregularly stenosed permeable walled artery under periodic body acceleration. Sharma et al.^57^ analyzed entropy generation effects on EMHD Jeffrey fluid flow over a vertical stretching surface.

The current physics of flow across a vertical stretching surface is expected to serve as the foundation for various medical science, engineering, and technology applications. The combined effect of physical characteristics may aid scientists in comprehending their findings. To the best of our knowledge, no effort has been yet made to perform the entropy generation minimization of higher-order endothermic/exothermic chemical reactions with activation energy on MHD mixed convective flow over a vertical stretching surface in the presence of thermophoresis and Brownian motion. Hence, the motivation from the above studies inspired us to perform this analysis. The following are some significant novel aspects included in this study: (1) to perform entropy generation minimization of higher-order endothermic/exothermic chemical reactions with activation energy, (2) to incorporate thermophoresis and Brownian motion effects along with the imposition of a time-dependent inclined magnetic field, (3) to add velocity, thermal, and concentration slips along with injection/suction effects. The current problem could help researchers use this method to solidify liquid metal from the mushy zone, build a metallic layer around a thermonuclear fusion-fission hybrid reactor, and produce drug delivery systems and gene therapy. The present study initiative is organized into six sections, as follows:

- The first section is an introduction, which describes the various physical quantities in this and other relevant studies.
- The model’s geometry and the flow’s governing equations are included in the second part, namely Mathematical Formulation.
- The third section comprises similarity transformation. Also, the given PDEs are converted to nonlinear coupled ODEs using these similarity transformations. This section introduces non-dimensional variables used in the present study to generate governing equation solutions.
- The fourth section is the numerical solution, which explains the numerical procedure used to solve the nonlinear coupled ODEs in which RK-4, along with the Newton Raphson shooting technique, are employed.
- The fifth section describes the minimization of entropy generation and Bejan number effects.
- Finally, there’s the section on Results and Graphical Analysis. The results are shown graphically in MATLAB, and the graphical results are then elaborated. The surface and contour plots are drawn to precisely analyze the flow parameter’s behavior.

## Mathematical formulation

**Figure 1:**
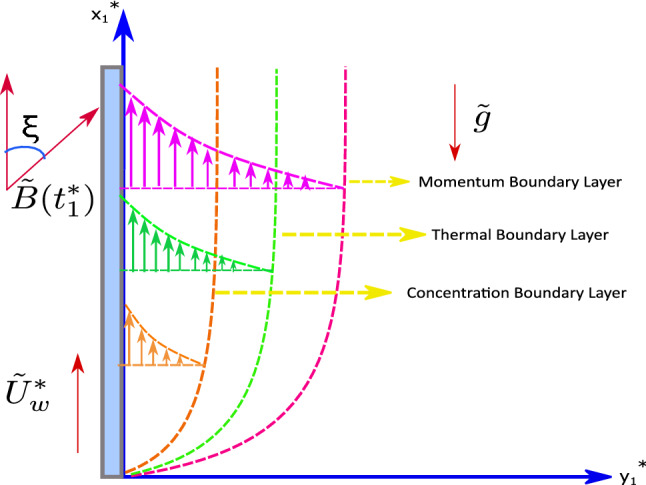
A pictorial representation of the model.

An unsteady, incompressible, laminar, viscous, electrically conducting MHD boundary layer flow across a stretching vertical sheet with viscous dissipation, thermophoresis, Brownian motion, Joule heating, and higher-order endothermic/exothermic chemical reaction is under consideration. The cartesian coordinate system is used with the $x_1^*$ and the $y_1^*$ axis. Within the fluid medium, the origin is considered fixed with ambient temperature $T_\infty ^*$, and the surface is kept at uniform temperature ${\tilde{T}}_w$. The surface concentration is kept uniform at ${\tilde{C}}_w$ whereas the ambient fluid concentration is $C_\infty ^*$. The stretching sheet velocity is

$$\begin{aligned} {\tilde{U}}_w^*={\tilde{p}}x_1^*(1-{\tilde{r}}t_1^*)^{-1}, \end{aligned}$$

along $x_1^*$ axis at time $t_1^*=0$, where ${\tilde{p}}$ and ${\tilde{r}}$ are constants. Here, ${\tilde{p}}$ represents the initial stretching rate, while $\frac{{\tilde{p}}}{(1-{\tilde{r}}t_1^*)}$ represents the effective stretching rate over time. An inclined magnetic field ${\tilde{B}}(t_1^*)$ with an acute angle $\xi$ is applied along the $x_1^*$-direction. The magnetic Reynold’s number is assumed to be very small $(Re \ll 1)$ in this study, so the induced magnetic field effect can be neglected. Figure 1 depicts the pictorial representation of the model. Based on the above assumptions and using the order of magnitude approach along with the usual Boussinesq’s approximation for the boundary layer, the governing equations are^58–60^:

1 $$\begin{aligned} {}&\frac{\partial {\tilde{u}}_1^*}{\partial x_1^*}+\frac{\partial {\tilde{v}}_1^*}{\partial y_1^*}=0{,} \end{aligned}$$

2 $$\begin{aligned}&\frac{\partial {\tilde{u}}_1^*}{\partial t_1^*}+{{\tilde{u}}_1^*}\frac{\partial {\tilde{u}}_1^*}{\partial x_1^*}+ {{\tilde{v}}_1^*}\frac{\partial {\tilde{u}}_1^*}{\partial y_1^*}={\tilde{\nu }}^*\frac{\partial ^2 {\tilde{u}}_1^*}{\partial y_1^{*2}}+{\tilde{g}}\beta ({\tilde{T}}_f-T_\infty ^*)+{\tilde{g}}\beta ^*({\tilde{C}}_f-C_\infty ^*)-\frac{{\tilde{\sigma }} {\tilde{B}}^2(t_1^*)}{{\tilde{\rho }}^*}sin^2\xi {{\tilde{u}}_1^*}{,} \end{aligned}$$

3 $$\begin{aligned}&\frac{\partial {\tilde{T}}_f}{\partial t_1^*}+{{\tilde{u}}_1^*}\frac{\partial {\tilde{T}}_f}{\partial x_1^*}+{{\tilde{v}}_1^*}\frac{\partial {\tilde{T}}_f}{\partial y_1^*}= \frac{{\tilde{k}}^*}{{\tilde{\rho }}^* {\tilde{C}}_p^*}\frac{\partial ^2 {\tilde{T}}_f}{\partial y_1^{*2}}+\frac{{\tilde{\nu }}^*}{{\tilde{C}}_p^*}\bigg (\frac{\partial {\tilde{u}}_1^*}{\partial y_1^*}\bigg )^2+\frac{{\tilde{\sigma }} {\tilde{B}}^2}{{\tilde{\rho }}^* {\tilde{C}}_p^*}({{\tilde{u}}_1^*})^2 sin^2\xi +{\tilde{\tau }}^*\bigg [\frac{{\tilde{D}}_t}{T_\infty ^*}\bigg (\frac{\partial {\tilde{T}}_f}{\partial y_1^*}\bigg )^2+{\tilde{D}}_b\frac{\partial {\tilde{C}}_f}{\partial y_1^*}\frac{\partial {\tilde{T}}_f}{\partial y_1^*}\bigg ]\nonumber \\&\quad +{\tilde{\beta }}_1^*\Gamma (t_1^*)({C}_f-C_\infty ^*)^N\bigg (\frac{{\tilde{T}}_f}{T_\infty ^*}\bigg )^m exp\bigg (\frac{-{\tilde{E}}_a}{{\tilde{k}}_1 {\tilde{T}}_f}\bigg ){,} \end{aligned}$$

4 $$\begin{aligned}&\frac{\partial {\tilde{C}}_f}{\partial t_1^*}+{{\tilde{u}}_1^*} \frac{\partial {\tilde{C}}_f}{\partial x_1^*}+{{\tilde{v}}_1^*} \frac{\partial {\tilde{C}}_f}{\partial y_1^*}={\tilde{D}}_b\frac{\partial ^2 {\tilde{C}}_f}{\partial y_1^{*2}}+\frac{{\tilde{D}}_t}{T_\infty ^*}\bigg (\frac{\partial ^2 {\tilde{T}}_f}{\partial y_1^{*2}}\bigg )-\Gamma (t_1^*)({C}_f-C_\infty ^*)^N\bigg (\frac{{\tilde{T}}_f}{T_\infty ^*}\bigg )^m exp\bigg (\frac{-{\tilde{E}}_a}{{\tilde{k}}_1 {\tilde{T}}_f}\bigg ){.} \end{aligned}$$

The boundary conditions subject to the flow are^58,61^:

5 $$\begin{aligned} {} \begin{aligned} {\tilde{u}}_1^*&={\tilde{U}}_w^*(x_1^*,t_1^*)+{\tilde{E}}{\tilde{\mu }}^*\frac{\partial {\tilde{u}}_1^*}{\partial y_1^*},~~~~~~~{\tilde{v}}_1^*={\tilde{V}}_w^*(t_1^*),~~~~~~~~~~~~~~~~~~~~~~~~ \\ {\tilde{T}}_f&={\tilde{T}}_w(x_1^*,t_1^*)+{\tilde{F}}\frac{\partial {\tilde{T}}_f}{\partial y_1^*} , ~~~~~~ {\tilde{C}}_f={\tilde{C}}_w(x_1^*,t_1^*)+{\tilde{G}}\frac{\partial {\tilde{C}}_f}{\partial y_1^*} ~ ~~~~~~ at ~~y_1^*=0{,} \end{aligned} \end{aligned}$$

and

6 $$\begin{aligned} {} {\tilde{u}}_1^*\rightarrow 0,~~~~~~~~~{\tilde{T}}_f\rightarrow T_\infty ^* , ~~~~~~ {\tilde{C}}_f\rightarrow C_\infty ^*~ ~~ ~~~~ at ~~y_1^*\rightarrow \infty {.} \end{aligned}$$

${\tilde{V}}_w^*$ is specified by

7 $$\begin{aligned} {} {\tilde{V}}_w^*=-\sqrt{\frac{{\tilde{\nu }}^* {\tilde{U}}_w^*}{x_1^*}}{\tilde{\chi }}(0){,} \end{aligned}$$

where, ${\tilde{V}}_w^* > 0$ demonstrates injection and ${\tilde{V}}_w^* < 0$ demonstrates suction.

In Eq. (5), ${\tilde{E}}={\tilde{E}}_0(1-{\tilde{r}}t_1^*)^{1/2}$, ${\tilde{F}}={\tilde{F}}_0(1-{\tilde{r}}t_1^*)^{1/2}$, ${\tilde{G}}={\tilde{G}}_0(1-{\tilde{r}}t_1^*)^{1/2}$ represent velocity, thermal and concentrations respectively with $E_0$, $F_0$, $G_0$, being their initial values. Also, $E_0=0$, $F_0=0$, $G_0=0$ corresponds to the no-slip boundary condition.

Further, it is assumed that

8 $$\begin{aligned} {} {\tilde{U}}_w^*=\frac{{\tilde{p}}x_1^*}{1-{\tilde{r}}t_1^*}, {\tilde{T}}_w=T_\infty ^*+\frac{{\tilde{q}}x_1^*}{1-{\tilde{r}}t_1^*}, {\tilde{C}}_w=C_\infty ^*+\frac{{\tilde{s}}x_1^*}{1-{\tilde{r}}t_1^*}{,} \end{aligned}$$

where ${\tilde{p}}>0,{\tilde{q}} \ge 0,{\tilde{r}} \ge 0,{\tilde{s}} \ge 0$ are constants and ${\tilde{r}}t_1^*<1$.

We consider ${\tilde{B}}={\tilde{B}}_0^*(1-{\tilde{r}}t_1^*)^{-1/2}~and~\Gamma (t_1^*)=\Gamma _0(1-{\tilde{r}}t_1^*)^{-1}$ where ${\tilde{B}}_0^*$ represents the magnetic field strength at $t_1^*=0$ and $\Gamma _0$ is a constant.

## Similarity transformations

The following similarity transformation is used to obtain the solution to the governing equations:

9 $$\begin{aligned} {} \eta =\bigg (\frac{{\tilde{U}}_w^*}{{\tilde{\nu }}^*x_1^*}\bigg )^{1/2}y_1^*,~ \psi ^*=({\tilde{\nu }}^*x_1^*{\tilde{U}}_w^*)^{1/2}{\tilde{\chi }}(\eta ),~ {\tilde{\zeta }}(\eta )=\frac{{\tilde{T}}_f-T_\infty ^*}{{\tilde{T}}_w-T_\infty ^*},~ {\tilde{\phi }}(\eta )=\frac{{\tilde{C}}_f-C_\infty ^*}{{\tilde{C}}_w-C_\infty ^*}{,} \end{aligned}$$

where $\psi ^*$ is the stream function that satisfies the continuity equation (1).

The velocity components are: ${\tilde{u}}_1^*=\frac{\partial \psi ^*}{\partial y_1^*}$ and ${\tilde{v}}_1^*=-\frac{\partial \psi ^*}{\partial x_1^*}$.

On calculating we have ${\tilde{u}}_1^*={\tilde{U}}_w^* {\tilde{\chi }}'(\eta )$ and ${\tilde{v}}_1^*=-\sqrt{\frac{{\tilde{p}}{\tilde{\nu }}^*}{(1-{\tilde{r}}t_1^*)}}{\tilde{\chi }}$.

The substitution of the similarity transformation introduced in Eq. (9) to the governing Eqs. (2)–(4), the following set of ODEs is obtained:

10 $$\begin{aligned} {}&{\tilde{\chi }}'''+{\tilde{\chi }}{\tilde{\chi }}''-({\tilde{\chi }}')^2-A\left( {\tilde{\chi }}'+\frac{1}{2}\eta {\tilde{\chi }}''\right) +Gr {\tilde{\zeta }}+ Gc {\tilde{\phi }}-M^2 sin^2\xi {\tilde{\chi }}'=0{,} \end{aligned}$$

11 $$\begin{aligned}{}&\frac{1}{Pr}{\tilde{\zeta }}''+{\tilde{\chi }}{\tilde{\zeta }}'-{\tilde{\chi }}'{\tilde{\zeta }}-A\left( {\tilde{\zeta }}+\frac{1}{2}\eta {\tilde{\zeta }}'\right) +Ec({\tilde{\chi }}'')^2+EcM^2 sin^2\xi ({\tilde{\chi }}')^2\nonumber \\&\quad +N_b{\tilde{\zeta }}'{\tilde{\phi }}'+N_t({\tilde{\zeta }}')^2+{\tilde{\lambda }}_1{\tilde{\sigma }}_1(1+\delta ^*{\tilde{\zeta }})^m{\tilde{\phi }}^N exp\bigg (\frac{-{\tilde{E}}^*}{1+\delta ^*{\tilde{\zeta }}}\bigg )=0{,} \end{aligned}$$

12 $$\begin{aligned}{}&\frac{1}{Sc}{\tilde{\phi }}''+{\tilde{\chi }}{\tilde{\phi }}'-{\tilde{\chi }}'{{\tilde{\phi }}}-A\left( {{\tilde{\phi }}}+\frac{1}{2}\eta {{\tilde{\phi }}}'\right) +\frac{N_t}{N_b Sc}{{\tilde{\zeta }}}''-{{\tilde{\sigma }}}_1(1+\delta ^*{{\tilde{\zeta }}})^m{{\tilde{\phi }}}^N exp\bigg (\frac{-{\tilde{E}}^*}{1+\delta ^*{{\tilde{\zeta }}}}\bigg )=0{.} \end{aligned}$$

The non-dimensional parameters used in the above equations are mentioned in Table 1.

The dimensional boundary conditions are reduced to the following non-dimensional boundary conditions:

13 $$\begin{aligned} {{\tilde{\chi }}}&=S,~ {{\tilde{\chi }}}'=1+S_v{{\tilde{\chi }}}''(0),~ {{\tilde{\zeta }}}=1+S_t{{\tilde{\zeta }}}'(0),~ {{\tilde{\phi }}}=1+S_c{{\tilde{\phi }}}'(0)~~at~~\eta =0,\nonumber \\ {{\tilde{\chi }}}'&\rightarrow 0,~ {{\tilde{\zeta }}} \rightarrow 0,~ {{\tilde{\phi }}} \rightarrow 0 ~~~~~ as~~~~\eta \rightarrow \infty {.} \end{aligned}$$

In Eq. (13), the injection is represented by $S \le 0$ whereas suction is represented by $S \ge 0$. Also,

$$\begin{aligned} S_v={\tilde{E}}_0 {{\tilde{\rho }}}^*\sqrt{{\tilde{p}}{\tilde{v}}_1^*},~~S_t={\tilde{F}}_0\sqrt{\frac{{\tilde{p}}}{{\tilde{v}}_1^*}},~~S_c={\tilde{G}}_0\sqrt{\frac{{\tilde{p}}}{{\tilde{v}}_1^*}} \end{aligned}$$.

**Table 1:** Non-dimensional parameter used in analysis.

$A=\frac{{\tilde{r}}}{{\tilde{p}}}$	$M={\tilde{B}}_0\sqrt{\frac{{{\tilde{\sigma }}}}{{{\tilde{\rho }}}^* {\tilde{p}}}}$	$Gr=\frac{{\tilde{g}} {{\tilde{\beta }}} x_1^*({\tilde{T}}_w-T_\infty ^*)}{{\tilde{U}}_w^{*2}}$	$Gc=\frac{{\tilde{g}}\beta ^*x_1^*({\tilde{C}}_w-C_\infty ^*)}{{\tilde{C}}_w^2}$
$Ec=\frac{{\tilde{U}}_w^{*2}}{{\tilde{C}}_p^*({\tilde{T}}_w-T_\infty ^*)}$	$Pr=\frac{{{\tilde{\mu }}}^* {\tilde{C}}_p^*}{{\tilde{k}}^*}$	$Sc=\frac{{{\tilde{\nu }}}^*}{{\tilde{D}}}$	${{\tilde{\sigma }}}_1=\frac{\Gamma _0(\tilde{C_w}-C_\infty ^*)^{N-2}}{{\tilde{p}}}$
$Re=\frac{{\tilde{U}}_w^* x_1^*}{{{\tilde{\nu }}}^*}$	$Br=\frac{{{\tilde{\mu }}}^*{\tilde{U}}_w^{*2}}{{\tilde{k}}^*({\tilde{T}}_w-T_\infty ^*)}$	$\Omega =\frac{({\tilde{T}}_w-T_\infty ^*)}{T_\infty ^*}$	$\Psi =\frac{({\tilde{C}}_w-C_\infty ^*)}{C_\infty ^*}$
$N_t=\frac{{{\tilde{\tau }}}^* {\tilde{D}}_t ({\tilde{T}}_w-T_\infty ^*)}{{{\tilde{\nu }}}^* T_\infty ^*}$	$N_b=\frac{{{\tilde{\tau }}}^* {\tilde{D}}_b ({\tilde{C}}_w-C_\infty ^*)}{{{\tilde{\nu }}}^*}$	${\tilde{E}}^*=\frac{{\tilde{E}}_a}{{\tilde{k}}_1 {\tilde{T}}_f}$	${{\tilde{\lambda }}}_1=\frac{{{\tilde{\beta }}}_1^* ({\tilde{C}}_w-C_\infty ^*)^2}{{{\tilde{\rho }}}^* {\tilde{C}}_p^* ({\tilde{T}}_w-T_\infty ^*)^2}$

**Figure 2:**
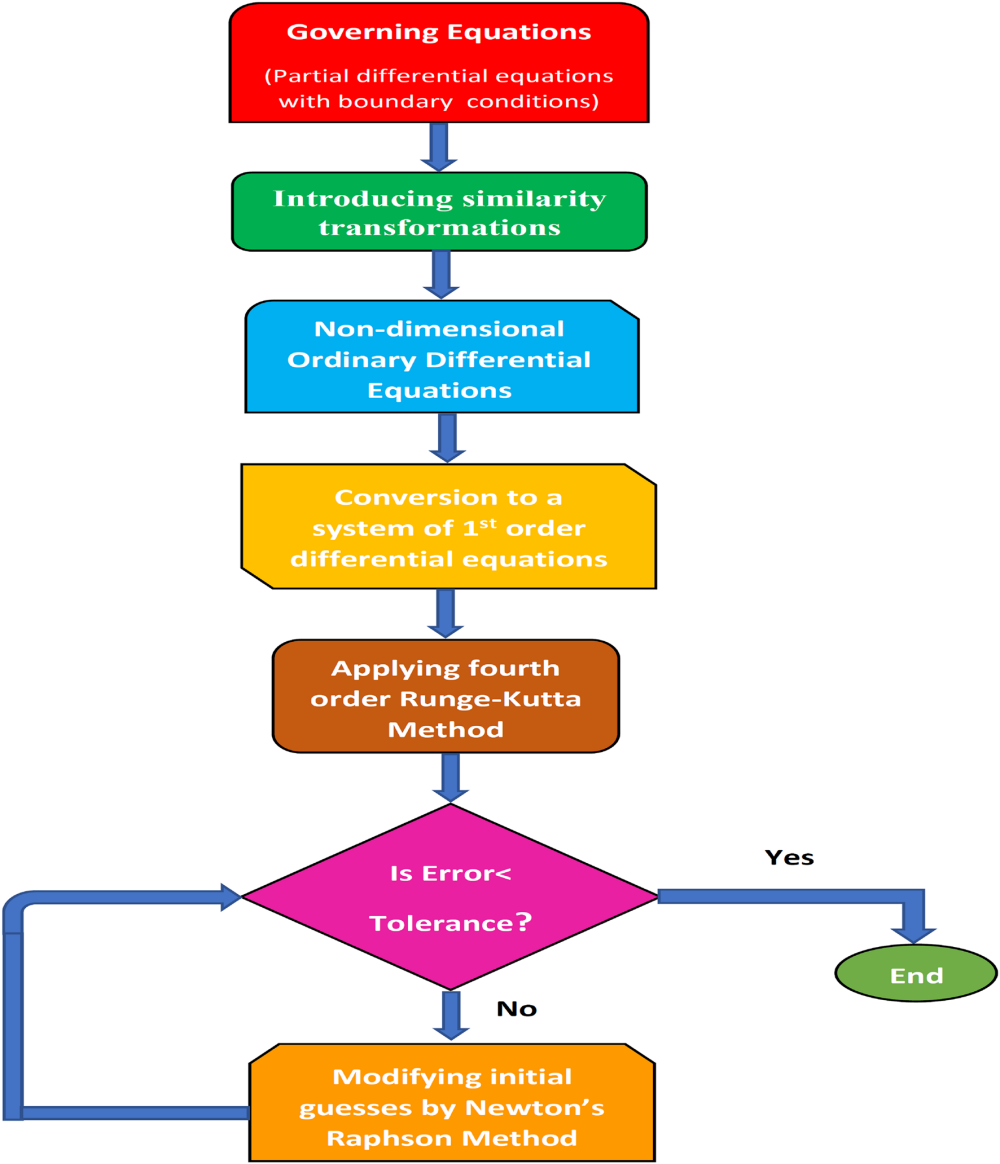
Flow chart illustrating solution methodology.

## Numerical solution

Equations (10)–(12) are the higher-order nonlinear coupled ordinary differential equation. To solve the system of coupled non-linear ODEs (10)–(12) with boundary conditions (13), the Newton Raphson shooting technique is employed in combination with the RK-4 algorithm. The boundary value problem of the physical model is first turned into an initial value problem. The system of Eqs. (10)–(12) comprises three differential equations out of which one is third-order, and the other two are second-order equations. Therefore, it cannot be solved until seven initial conditions are specified. However, initially, there are only four conditions defined as given in Eq. (13). For obtaining the solution, the conditions ${{\tilde{\chi }}}'(\infty )=0$, ${{\tilde{\zeta }}}(\infty )=0$, and ${{\tilde{\phi }}}(\infty )=0$ are replaced by ${{\tilde{\chi }}}''(0)=l_1$, ${{\tilde{\zeta }}}'(0)=l_2$, and ${{\tilde{\phi }}}'(0)=l_3$ (initial guesses). Furthermore, the $\eta _{\infty }$ should have a finite upper bound. The solution is then calculated using the RK fourth-order approach. Finally, the computed solution will converge if the residuals are less than the error tolerance ($10^{-6}$). The Newton’s method is used to modify the original guesses if the computed solution fails to meet the convergence condition. The solution procedure is depicted with the help of a flowchart in Fig. 2.

The governing Eqs. (10)–(12) are the nonlinear coupled ordinary differential equation. For solving, these are converted into a system of first order differential equations. Let

14 $$\begin{aligned} {\left\{ \begin{array}{ll} {{\tilde{\chi }}}=f_1~, ~~~~~ {{\tilde{\chi }}}'=f_2~, ~~~~~{{\tilde{\chi }}}''=f_3,\\ {{\tilde{\zeta }}}=f_4 ~,~~~~~~ {{\tilde{\zeta }}}'=f_5,\\ {{\tilde{\phi }}}=f_6~,~~~~~~{{\tilde{\phi }}}'=f_7. \end{array}\right. } \end{aligned}$$

Therefore, on introducing these new variables the Eqs. (10)–(12) transformed into the following system-

15 $$\begin{aligned} {\left\{ \begin{array}{ll} f_1'=f_2,\\ f_2'=f_3,\\ f_3'=-f_1f_3+(f_2)^2+A\bigg (f_2+\frac{1}{2} \eta f_3\bigg )-Grf_4-Gcf_6+M^2 sin^2 \xi f_2,\\ f_4'=f_5,\\ f_5'=Pr\bigg [-f_1f_5+f_2f_4+A \left( f_4+\frac{1}{2} \eta f_5 \right) -Ec \left( (f_3)^2+M^2 sin^2 \xi (f_2)^2 \right) -N_b f_5 f_7 -N_t (f_5)^2 -{{\tilde{\lambda }}}_1{{\tilde{\sigma }}}_1(1+\delta ^* f_4)^m (f_6)^N exp\bigg (\frac{-{\tilde{E}}^*}{1+\delta ^* f_4}\bigg )\bigg ],\\ f_6'=f_7,\\ f_7'=Sc\bigg [-f_1f_7+f_2f_6+A\left( f_6+\frac{1}{2} \eta f_7\right) +{{\tilde{\sigma }}}_1(1+\delta ^* f_4)^m (f_6)^N exp\bigg (\frac{-{\tilde{E}}^*}{1+\delta ^* f_4}\bigg )\bigg ]-\frac{N_t}{N_b}(f_5')^2.\\ \end{array}\right. } \end{aligned}$$

and the boundary conditions transformed from the equation (13) are-

16 $$\begin{aligned} {\left\{ \begin{array}{ll} f_1(0)=S~ ,~~f_2=1+S_v f_3(0)~,\\ f_4=1+S_t f_5(0)~, ~~f_6=1+S_c f_7(0), \\ f_2 \rightarrow 0 , ~~~~~~~~ f_4 \rightarrow 0 , ~~~~~~~~~ f_6 \rightarrow 0. \end{array}\right. } \end{aligned}$$

## Entropy generation

The entropy of a system is a broad attribute that changes as mass and energy are exchanged. The overall entropy of a system made up of numerous processes is equal to the sum of the entropies produced by each process. The entropy generation rate due to the exchange of momentum, energy, and mass explains the irreversibility of MHD mixed convective flow over a vertical stretching surface with Joule effect, thermophoresis, Brownian motion, viscous dissipation and higher-order endothermic/exothermic chemical with activation energy. The volumetric entropy generation is defined as^51,52^:

17 $$\begin{aligned} E_g=\underbrace{\frac{{\tilde{k}}^*(\nabla {\tilde{T}}_f)^2}{T_\infty ^{*2}}}_\text {Thermal irreversibility}+\underbrace{\frac{{{\tilde{\mu }}}^*}{T_\infty ^*}{\tilde{F}}^*}_\text {Viscous irreversibility}+\underbrace{\frac{{{\tilde{\sigma }}} {\tilde{B}}^2}{T_\infty ^{*2}} sin^2 \xi u_1^{*2}}_\text {Joule heating irreversibility}+\underbrace{\frac{{\tilde{R}}{\tilde{D}}_b(\nabla {\tilde{C}}_f)^2}{C_\infty ^*}+\frac{{\tilde{R}}{\tilde{D}}_b}{T_\infty ^*}(\nabla {\tilde{T}}_f)(\nabla {\tilde{C}}_f)}_\text {Diffusive irreversibility}{,} \end{aligned}$$

where ${\tilde{F}}^*$ represents viscous dissipation.

Therefore, the entropy generation rate due to the exchange of momentum, energy, and mass is given as:

18 $$\begin{aligned} {} E_g=\frac{{\tilde{k}}^*}{T_\infty ^{*2}}\bigg (\frac{\partial {\tilde{T}}_f}{\partial y_1^*}\bigg )^2+\frac{{{\tilde{\mu }}}^*}{T_\infty ^*}\bigg (\frac{\partial u_1^*}{\partial y_1^*}\bigg )^2+\frac{{{\tilde{\sigma }}} {\tilde{B}}^2}{T_\infty ^{*2}} sin^2 \xi u_1^{*2}+\frac{{\tilde{R}}{\tilde{D}}_b}{C_\infty ^*}\bigg (\frac{\partial {\tilde{C}}_f}{\partial y_1^*}\bigg )^2+\frac{{\tilde{R}}{\tilde{D}}_b}{T_\infty ^*}\bigg (\frac{\partial {\tilde{C}}_f}{\partial y_1^*}\bigg )\bigg (\frac{\partial {\tilde{T}}_f}{\partial y_1^*}\bigg ){.} \end{aligned}$$

On applying the similarity transformation given in Eq. (9),

19 $$\begin{aligned} E_g= & {} \frac{{\tilde{k}}^*}{T_\infty ^{*2}}({\tilde{T}}_w-T_\infty ^*)^2\bigg (\frac{{\tilde{U}}_w^*}{{{\tilde{\nu }}}^*x_1^*}\bigg )({{\tilde{\zeta }}}')^2+\frac{{{\tilde{\mu }}}^*}{T_\infty ^*}\bigg (\frac{{\tilde{U}}_w^{*3}}{{{\tilde{\nu }}}^*x_1^*}\bigg )({{\tilde{\chi }}}'')^2+\frac{{{\tilde{\sigma }}} {\tilde{B}}_0^2}{T_\infty ^{*2}(1-{\tilde{r}}t_1^*)}{\tilde{U}}_w^{*2} sin^2 \xi ({{\tilde{\chi }}}')^2+\frac{{\tilde{R}}{\tilde{D}}_b}{C_\infty ^*}({\tilde{C}}_w-C_\infty ^*)^2\bigg (\frac{{\tilde{U}}_w^*}{{{\tilde{\nu }}}^*x_1^*}\bigg )(\tilde{\phi '})^2 +\frac{{\tilde{R}}{\tilde{D}}_b}{T_\infty ^*}({\tilde{C}}_w-C_\infty ^*)({\tilde{T}}_w-T_\infty ^*)\bigg (\frac{{\tilde{U}}_w^*}{{{\tilde{\nu }}}^*x_1^*}\bigg )\tilde{\phi '}{{\tilde{\zeta }}}'{.} \end{aligned}$$

The dimensionless entropy generation number can be described as the ratio of the characteristics entropy generation rate to the actual entropy generation rate.

20 $$\begin{aligned} {} N_s=Re({{\tilde{\zeta }}}')^2+\frac{Re Br}{\Omega }({{\tilde{\chi }}}'')^2+\frac{Re Br M^2}{\omega }({{\tilde{\chi }}}')^2+Re \Lambda \bigg (\frac{\Psi }{\Omega }\bigg )^2(\tilde{\phi '})^2+\frac{Re \Psi \Lambda }{\Omega }\tilde{\phi '}\tilde{\zeta '}{.} \end{aligned}$$

Be depicts the ratio of irreversibility owing to heat transfer and total irreversibility owing to heat transfer and fluid friction. In mathematical form, it is described as:

21 $$\begin{aligned} {} Be=\frac{N}{N_s}=\frac{Re({{\tilde{\zeta }}}')^2}{Re({{\tilde{\zeta }}}')^2+\frac{Re Br}{\Omega }({{\tilde{\chi }}}'')^2+\frac{Re Br M^2}{\omega }({{\tilde{\chi }}}')^2+Re \Lambda \bigg (\frac{\Psi }{\Omega }\bigg )^2(\tilde{\phi '})^2+\frac{Re \Psi \Lambda }{\Omega }\tilde{\phi '}\tilde{\zeta '}}{.} \end{aligned}$$

## Results and graphical analysis

The present study discusses the entropy generation effects of higher-order endothermic/exothermic chemical reactions on MHD mixed convective flow across a vertical stretching surface with Joule heating, thermophoresis, Brownian motion, and viscous dissipation. The influence of velocity, thermal, and concentration slip is also investigated. The impact of the flow parameters identified in the problem such as Hartmann number (M), Grashof number (Gr), solutal grashof number (Gc), velocity slip ($S_v$), suction parameter (S), inclination parameter ($\xi$), Prandtl number (Pr), thermophoresis parameter ($N_t$), Eckert number (Ec), endothermic/exothermic reaction parameter (${{\tilde{\lambda }}}_1$), Brownian motion parameter ($N_b$), activation energy parameter (${\tilde{E}}^*$), thermal slip ($S_t$), order of chemical reaction (N), Schmidt number (Sc), concentration slip ($S_c$), and chemical reaction parameter (${{\tilde{\sigma }}}_1$) is explored on the entropy generation ($N_s$), Bejan number (Be), velocity profile (${{{\tilde{\chi }}}}'(\eta )$), temperature profile (${{{\tilde{\zeta }}}}(\eta )$), and the concentration profile (${{{\tilde{\phi }}}}(\eta )$) to gain a physical understanding of the problem. For numerical results, some default values for the parameters are depicted in Table 2. These values are considered default unless mentioned in the relevant graphs.

**Table 2:** Default values of emerging parameters.

M	Gr	Gc	$\xi$	S	$S_{\nu }$	${\tilde{\lambda }}_{1}$	$\delta ^{*}$	${\tilde{\sigma }}_{1}$	$\tilde{E}^{*}$	$S_{t}$	$S_{c}$	Pr	$\hbox {N}_{\mathrm{b}}$	$\hbox {N}_{\mathrm{t}}$	Ec	Sc	m
3	2	2	$\frac{\pi }{4}$	0.5	1.5	0.2	0.5	0.4	0.5	1.0	0.5	7	0.5	0.5	0.5	0.6	0.5

**Figure 3:**
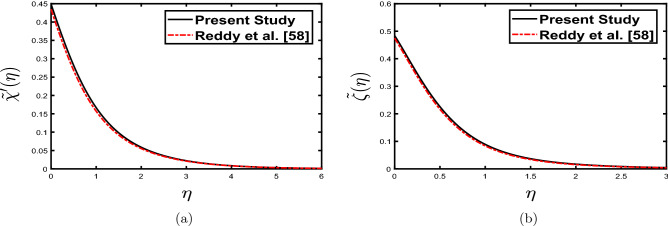
Comparative analysis of (**a**) velocity profile ${{\tilde{\chi }}}'(\eta )$ for M = 3, (**b**) temperature profile ${{\tilde{\zeta }}}(\eta )$ for Pr = 7.

The effect of inclination of the surface, permeability, heat source, and radiation parameter in^58^ and the influence of thermophoresis, Brownian motion, and higher-order endothermic/exothermic reaction with activation energy in the present work is neglected to validate the current results with Reddy et al.^58^. Figure 3 depict the present work’s velocity and temperature profile with the study done by Reddy et al.^58^. Further, the results are compared with the available results of Sharma and Gandhi^62^. Table 3 shows the comparison of ${{\tilde{\chi }}}''(0),-{{\tilde{\zeta }}}'(0),-{{\tilde{\phi }}}'(0)$ for^62^ and the present work. The current results are in good agreement (under specific limiting conditions), clearly demonstrating the accuracy of the computed results.

**Table 3:** Comparsion of present results with Sharma and Gandhi^62^ for ${{\tilde{\chi }}}''(0),-{{\tilde{\zeta }}}'(0),-{{\tilde{\phi }}}'(0)$.

M	Gr	Gc	Ec	Pr	Sc	$\chi ^{\prime \prime }(0)$ (Sharma and Gandhi^62^)	$\chi ^{\prime \prime }(0)$ (Present results)	$-{{{\tilde{\zeta }}}} '(0)$ (Sharma and Gandh^62^)	$-{{{\tilde{\zeta }}}} '(0)$ (Present results)	$-{{\tilde{\phi }}} '(0)$ (Sharma and Gandh^62^)	$-{{\tilde{\phi }}} '(0)$ (Present results)
3	2	2	0.5	7	0.6	− 0.46690320	− 0.46688594	0.65314058	0.65299325	0.55094993	0.55099047
4	2	2	0.5	7	0.6	− 0.52048934	− 0.52048977	0.67938705	0.67938637	0.52661434	0.52662259
3	3	2	0.5	7	0.6	− 0.45242293	− 0.45237258	0.62944823	0.62922132	0.55644428	0.55663530
3	2	3	0.5	7	0.6	− 0.42929373	− 0.42922739	0.57873245	0.57815509	0.56975363	0.56985386
3	2	2	0.75	7	0.6	− 0.45703607	− 0.45701714	0.54294834	0.54280225	0.55523360	0.55529090
3	2	2	0.5	9	0.6	− 0.46894612	− 0.46892418	0.67493590	0.67474532	0.55006986	0.55011481
3	2	2	0.5	7	0.78	− 0.47351894	− 0.47351050	0.66698164	0.66690862	0.61776205	0.61778105

**Figure 4:**
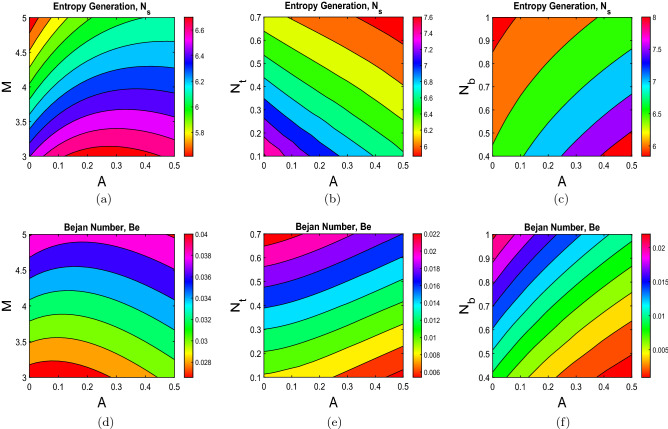
(**a**) $N_s$ versus M, (**b**) $N_s$ versus $N_t$, (**c**) $N_s$ versus $N_b$, (**d**) Be versus M, (**e**) Be versus $N_t$, and (**f**) Be versus $N_b$.

A contour plot is a 2-D representation of the surface in which similar-responding points are linked to generate contour lines with constant responses. The contour plots help determine the desired response values and operating circumstances. Figure 4 represents the contour plots for entropy generation ($N_s$) and Bejan number (Be). The influence of Hartmann number (M) on entropy generation ($N_s$) and Bejan number (Be) is shown via contours in Fig. 4a,d. It is noticed that as M increases, entropy drops while Be increases. Figure 4b,e illustrate the contour plots depicting the influence of thermophoresis parameter ($N_t$) on entropy generation ($N_s$) and Bejan number (Be) respectively. With an increase in $N_t$ values, entropy decreases. However, an increment in Bejan number is investigated. Figure 4c,f highlight the contours depicting the influence of Brownian motion parameter ($N_b$) on entropy generation ($N_s$) and Bejan number (Be). As the $N_b$ values rise, so does Be. On the other hand, the $N_s$ is declining.

**Figure 5:**
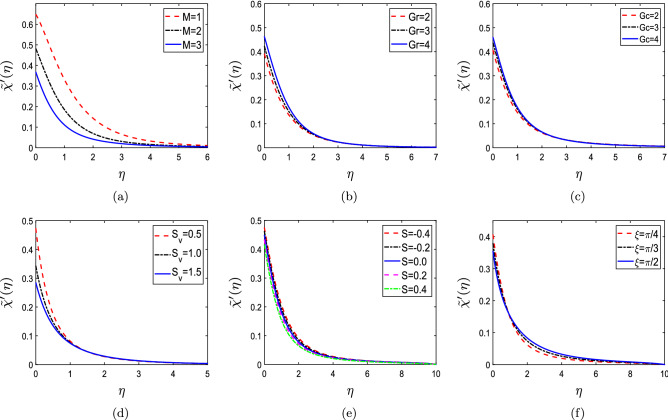
Non-dimensional velocity profiles for (**a**) Hartmann number (M), (**b**) Grashof number (Gr), (**c**) solutal Grashof number (Gc), (**d**) velocity slip ($S_v$), (**e**) suction parameter (S), and (**f**) inclination parameter ($\xi$).

The effect of different flow parameters namely Hartmann number (*M*), Grashof number (*Gr*), solutal Grashof number (*Gc*), velocity slip ($S_v$), suction parameter (*S*), inclination parameter ($\xi$) on dimensionless velocity $\chi '(\eta )$ is shown in Fig. 5. The velocity profile $\chi '(\eta )$ for various values of M is shown in Fig. 5a. The velocity profiles show deterioration as M increases from 3 to 5. The Lorentz force, thus generated, opposes the flow and retards the fluid’s velocity with the increment in values of M. The velocity profile $\chi '(\eta )$ for Gr and Gc are shown in Fig. 5b,c. The Gr is the proportion of buoyant and viscous forces in a fluid layer. Since the viscous forces become less dominating as the value of Gr enhances, the resistance to flow diminishes, and fluid flow velocity rises. There is an abrupt increase in velocity near the wall and afterward descends uniformly towards zero. The velocity profile for the solutal Grashof number Gc also increases with increasing Gc values. Figure 5d shows the profiles for non-dimensional velocity for $S_v$. The velocity profile deteriorates with increasing values of $S_v$. The momentum boundary layer rises as we increase the values of $S_v$, yet surface velocity exhibits a declining trend. This mechanism occurs because the fluid’s velocity is reduced due to the stretching velocity partially transferring the disturbance caused by frictional retardation between the surface and the fluid’s particles. Thus, the velocity profile drops. The non-dimensional velocity profile $\chi '(\eta )$ for S is depicted by Fig. 5e. With rising values of S, the velocity profiles decline slightly. The heated fluid is propelled towards the wall due to the considerable influence of viscosity, where buoyant forces can interfere to retard the fluid. Figure 5f expresses that increment in $\xi$ results in the decrement in non-dimensional velocity because the magnetic field strength enhances with the increment in the aligned angle. An opposite flow to force is generated due to this enhanced magnetic field, known as the Lorentz force. This generated force thus becomes a barrier in the fluid’s path. Hence, a decrease in $\chi '(\eta )$ is analyzed.

**Figure 6:**
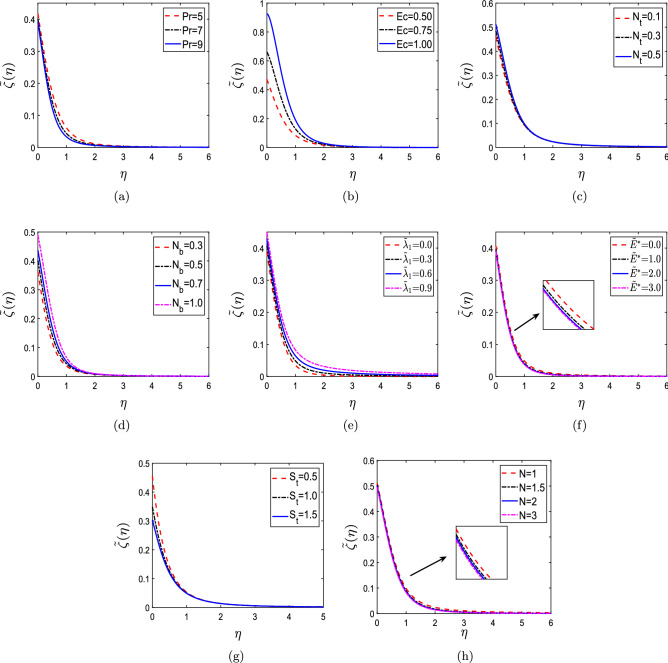
Non-dimensional temperature profiles for (**a**) Prandtl number (Pr), (**b**) Eckert number (Ec), (**c**) thermophoresis parameter ($N_t$), (**d**) Brownian motion parameter ($N_b$), (**e**) endothermic/exothermic reaction parameter (${{\tilde{\lambda }}}_1$), (**f**) activation energy parameter (${\tilde{E}}^*$), (g) thermal slip ($S_t$), and (**h**) order of chemical reaction (N).

The different parameters’ impact such as Prandtl number (Pr), Eckert number (Ec), thermophoresis parameter ($N_t$), Brownian motion parameter ($N_b$), endothermic/exothermic reaction parameter (${{\tilde{\lambda }}}_1$), activation energy parameter (${\tilde{E}}^*$), thermal slip ($S_t$), and order of chemical reaction (N) on temperature profile ${{{\tilde{\zeta }}}}(\eta )$ is highlighted in Fig. 6. Figure 6a highlights the influence of Pr on ${{{\tilde{\zeta }}}}(\eta )$. The temperature profile diminishes as Pr increases because Pr regulates the relative thickness of the thermal and momentum boundary layers. Because thermal conductivity increases as Pr decreases, heat diffusion from the heated surface happens faster for small Pr values than for large Pr values. The non-dimensional temperature profiles ${{{\tilde{\zeta }}}}(\eta )$ for different Ec values are shown in Fig. 6b. The temperature profiles ${{\tilde{\zeta }}}(\eta )$ rise with an increment in Ec as the internal energy increases. Ec is the relationship between the flow’s kinetic energy and the heat transfer’s enthalpy driving force. Figure 6c,d demonstrate the impact of $N_t$ and $N_b$ on dimensionless temperature profile ${{{\tilde{\zeta }}}}(\eta )$. The purpose of Fig. 6c is to show how $N_t$ affects the thermal field. Higher values of the thermophoretic parameter ($N_t$) are shown to result in elevated temperature profiles in the boundary layer region. This results from the fact that particles close to a hot surface produce a thermophoretic force that aids particle disintegration away from the fluid regime, which increases temperature boundary layer thicknesses. Figure 6d shows that a temperature rise is perceived for $N_b$. The random movement of suspended particles in the base fluid, known as Brownian motion, is more impacted by the fluid’s quickly moving atoms or molecules. It is important to note that Brownian motion is connected to particle size and that these particles frequently take the shape of aggregates or agglomerates. Brownian motion is very weak for massive particles, and the parameter ($N_b$) will have the lowest values. As the values of the Brownian motion parameter ($N_b$) increase, the temperature profiles in the boundary layer region increase. Figure 6e portrays the influence of ${{\tilde{\lambda }}}_1$ on ${{{\tilde{\zeta }}}}(\eta )$. An increment in the temperature profiles is observed with increasing values of ${{\tilde{\lambda }}}_1$, and the results obtained are consistent with that of^9^. Figure 6f shows the influence of ${\tilde{E}}^*$ on ${{{\tilde{\zeta }}}}(\eta )$. The temperature profiles improve for higher elevations of ${\tilde{E}}^*$ due to the generative reaction. The effect of $S_t$ on ${{{\tilde{\zeta }}}}(\eta )$ is depicted in Fig. 6g. There is a declination in temperature profiles with increasing values of $S_t$ since the thickness of the thermal boundary layer reduces. As a result, non-dimensional temperature profiles are diminished. Figure 6h shows the impact of N on ${{{\tilde{\zeta }}}}(\eta )$. A reduction in ${{{\tilde{\zeta }}}}(\eta )$ is seen with rising values of N. The obtained results show a good agreement with that of ^8^.

**Figure 7:**
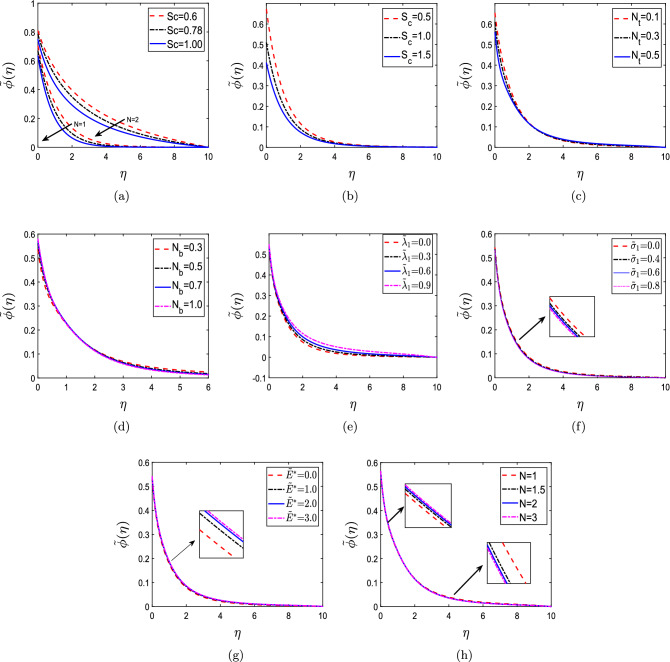
Non-dimensional concentration profiles for (**a**) Schmidt number (Sc), (**b**) concentration slip ($S_c$), (**c**) thermophoresis parameter ($N_t$), (**d**) Brownian motion parameter ($N_b$), (**e**) endothermic/exothermic reaction parameter (${{\tilde{\lambda }}}_1$), (**f**) chemical reaction parameter (${{\tilde{\sigma }}}_1$), (**g**) activation energy parameter (${\tilde{E}}^*$), and (**h**) order of chemical reaction (N).

The effect of several parameters on non-dimensional concentration ${{{\tilde{\phi }}}}(\eta )$, including Schmidt number (Sc), concentration slip ($S_c$), thermophoresis parameter ($N_t$), Brownian motion parameter ($N_b$), endothermic/exothermic reaction parameter (${{\tilde{\lambda }}}_1$), chemical reaction parameter (${{\tilde{\sigma }}}_1$), activation energy parameter (${\tilde{E}}^*$), and order of chemical reaction (N) is depicted in Fig. 7. Figure 7a shows the impact of Sc on non-dimensional concentration ${{{\tilde{\phi }}}}(\eta )$. The concentration profiles show declination with increment in the values of Sc for both N = 1 and N = 2. Sc refers to fluid flows simultaneously undergoing both momentum and mass diffusion convection processes. When the Schmidt number is large enough, momentum diffusion takes precedence over mass diffusion, and when it is small, mass diffusion takes precedence over momentum diffusion. The mass transfer boundary layer thickness is lesser than the hydrodynamic boundary layer thickness with increasing values of Schmidt number. Therefore, a decrease in non-dimensional concentration profiles ${{{\tilde{\phi }}}}(\eta )$ is investigated. Figure 7b represents the influence of $S_c$ on non-dimensional concentration ${{{\tilde{\phi }}}}(\eta )$. The concentration profile decreases as the value of $S_c$ increases, which is consistent with the results of ^58^. It is because slip essentially slows down fluid motion, which ultimately manifests as a reduction in net molecular mobility. Therefore, decreased molecular mobility causes mass fraction fields to decrease. The concentration slip parameter can likely control the mass transport phenomenon as the velocity slip parameter, and the thermal slip parameter can regulate the momentum and temperature inside the flow. In conclusion, the concentration boundary layer can be controlled up to the appropriate levels by modifying the concentration slip parameters. Figure 7c,d depicts the influence of $N_t$ and $N_b$ on non-dimensional concentration ${{{\tilde{\phi }}}}(\eta )$. The concentration in a narrow region near the surface is reduced when the thermophoretic parameter is increased. This phenomenon shows particles being ‘pushed’ out of the heated boundary layer into the colder free stream zone. This behavior changes when we proceed away from the surface into the free stream, and rising $N_t$ raises the concentration. On the other hand, the solute boundary layer diminishes as there is an increment in $N_b$. Particle mobility is aided by increasing $N_b$, which causes the boundary layer to warm, causing particles to move away from the surfaces within the inactive fluid. As a result, solute particle deposition away from the surface rises, causing concentration profiles to fall. The effect of ${{\tilde{\lambda }}}_1$ and ${{\tilde{\sigma }}}_1$ on non-dimensional concentration ${{\tilde{\phi }}}(\eta )$ is demonstrated by Fig. 7e,f. It is found that the non-dimensional concentration ${{{\tilde{\phi }}}}(\eta )$ enhances with increase in the value of ${{\tilde{\lambda }}}_1$. Increasing ${{\tilde{\sigma }}}_1$, on the other hand, causes the mass transfer boundary layer to thicken. It has been discovered that increasing the reaction rate constant results in an improvement in the factor ${{\tilde{\sigma }}}_1(1+\delta ^*{{\tilde{\zeta }}})^m exp\bigg (\frac{-{\tilde{E}}^*}{1+\delta ^*{{\tilde{\zeta }}}}\bigg )$. As a result, a destructive chemical reaction occurs, and the concentration profiles ${{{\tilde{\phi }}}}(\eta )$ diminishes. Figure 7g highlights the impact of ${\tilde{E}}^*$ on non-dimensional concentration ${{{\tilde{\phi }}}}(\eta )$. The definition of activation energy is that it is the least amount of energy necessary to initiate a reaction. It is discovered that higher activation energy causes a decrease in the reaction rate constant, which ultimately causes the chemical reaction to slow down. Additionally, the concentration profiles show enhancement. Figure 7h portrays the impact of N on non-dimensional concentration ${{{\tilde{\phi }}}}(\eta )$. There is an inclination in ${{{\tilde{\phi }}}}(\eta )$ with increasing values of N in a narrow region near the surface. However, it changes its behavior in the boundary layer and shows the opposite trend.

### Skin-friction coefficient ($C_f^*$), Nusselt number ($Nu_x^*$) and Sherwood number ($Sh_x^*$) results

Flow resistance directly correlates with the fluid flow rate and determines the physiological features of the flow. One of the physical quantities that influence the flow of fluid is shear-stress. The shear-stress expression in mathematical form is

$$\begin{aligned} {{\tilde{\tau }}}_w^*={{\tilde{\mu }}}^* \bigg (\frac{\partial {\tilde{u}}_1^*}{\partial y_1^*}\bigg )_{y_1^*=0} \end{aligned}$$

Therefore,

22 $$\begin{aligned} {} C_f^*=\frac{{{\tilde{\tau }}}_w^*}{({{\tilde{\rho }}}^* {\tilde{U}}_w^{*2})/2} \end{aligned}$$

To estimate and understand the heat transfer, the $Nu_x^*$, which is the ratio of convective to the conductive thermal transfer in the fluid across the boundary is calculated whose general expression is

23 $$\begin{aligned} {} Nu_x^*=\frac{x_1^*{\tilde{q}}_w^*}{{\tilde{k}}^*({\tilde{T}}_w-T_\infty ^*)}, \end{aligned}$$

where $${\tilde{q}}_w^*=-{\tilde{k}}^*\bigg (\frac{\partial {\tilde{T}}_f}{\partial y_1^*}\bigg )_{y_1^*=0}$$.

The ratio of the mass transfer due to convection to the diffusive mass rate (called Sherwood number) is

24 $$\begin{aligned} {} Sh_x^*=\frac{{\tilde{m}}_w^* x_1^*}{{{\tilde{\rho }}}^* {\tilde{D}} ({\tilde{C}}_w-C_\infty ^*)} \end{aligned}$$

where $${\tilde{m}}_w^*=-{{\tilde{\rho }}}^* {\tilde{D}}\bigg (\frac{\partial {\tilde{C}}_f}{\partial y_1^*}\bigg )_{y_1^*=0}$$.

Expressing in dimensionless form, we have

25 $$\begin{aligned} {} C_f^* = \frac{1}{2} Re_x^{-1/2} {{\tilde{\chi }}}''(0),~ Nu_x^* = - Re_x^{1/2} {{\tilde{\zeta }}}'(0),~ Sh_x^* = - Re_x^{1/2} {{\tilde{\phi }}}'(0) \end{aligned}$$

Tables 4 and 5 describe the values of ${{\tilde{\chi }}}''(0)$, $-{{\tilde{\zeta }}}'(0)$, $-{{\tilde{\phi }}}'(0)$ for contrasting values of velocity slip ($S_v$), thermal slip ($S_t$) and concentration slip ($S_c$) for A=0 and A=0.5, respectively. A decrement in skin-friction coefficient ($C_f^*$) is observed with increment in thermal slip ($S_t$) and concentration slip ($S_c$) whereas it increases with velocity slip ($S_v$). With an increment in thermal slip ($S_t$), the Nusselt number ($Nu_x^{*}$) declines whereas with increment in velocity slip ($S_v$) and concentration slip ($S_c$) it enhances. As the value of velocity slip ($S_v$), thermal slip ($S_t$) and concentration slip ($S_c$) is increased, a decrement in Sherwood number ($Sh_x^*$) is analyzed. The Nusselt number ($Nu_x^*$), and the Sherwood number ($Sh_x^*$) increase for velocity slip ($S_v$), thermal slip ($S_t$) and concentration slip ($S_c$) as the value of unsteadiness parameter A varies from 0 to 0.5. In contrast, the skin-friction coefficient ($C_f^*$) decreases for for velocity slip ($S_v$), thermal slip ($S_t$) and concentration slip ($S_c$) as the value of unsteadiness parameter A varies from 0 to 0.5.

**Figure 8:**
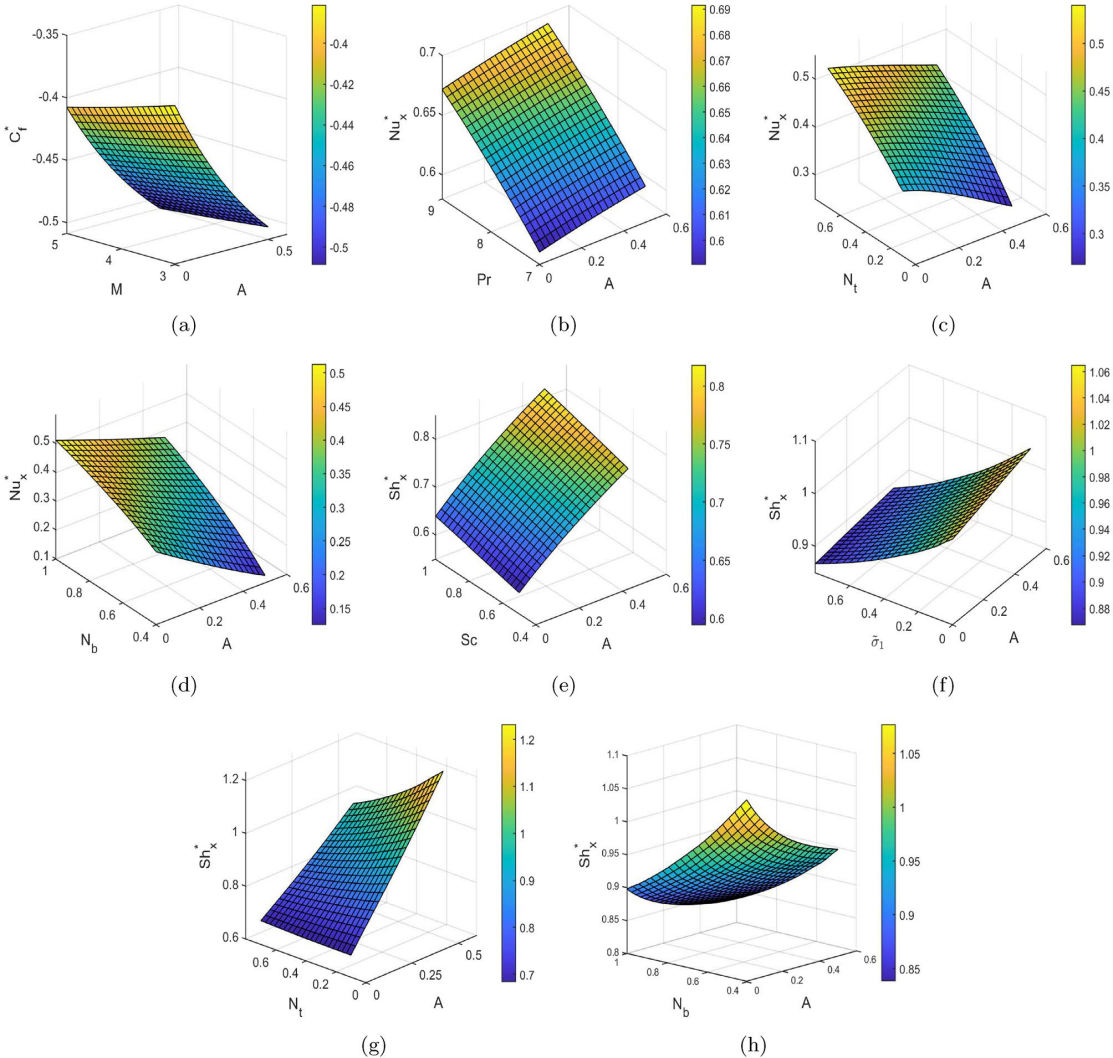
(**a**) $C_f^*$ versus M and A, (**b**) $Nu_x^*$ versus Pr and A, (**c**) $Nu_x^*$ versus $N_t$ and A, (**d**) $Nu_x^*$ versus $N_b$ and A, (**e**) $Sh_x^*$ versus Sc and A, (**f**) $Sh_x^*$ versus ${{\tilde{\sigma }}}_1$ and A, (**g**) $Sh_x^*$ versus $N_t$ and A, and (**h**) $Sh_x^*$ versus $N_b$ and A.

Surface plots are three-dimensional data visualizations. Surface plots demonstrate a functional relationship between a dependent variable and two independent variables rather than individual data points. Figure 8 represents surface plots for skin-friction coefficient ($C_f^*$), Nusselt number ($Nu_x^*$), and Sherwood number ($Sh_x^*$) for different flow parameters. The influence of Hartmann number (M) on skin-friction coefficient ($C_f^*$) is depicted in Fig. 8a. Skin-friction coefficient ($C_f^*$) decreases with increment in Hartmann number (M) as well unsteadiness parameter (A). Figure 8b shows the effect of Prandtl number (Pr) on Nusselt number ($Nu_x^*$). It is analyzed that there is an increase in the value of Nusselt number ($Nu_x^*$) with increment in both Prandtl number (Pr) as well as unsteadiness parameter (A). Figure 8c,d highlights the influence of thermophoresis ($N_t$) and Brownian motion ($N_b$) parameters on Nusselt number ($Nu_x^*$). It can be viewed that there is an increment in Nusselt number ($Nu_x^*$) with increasing values of both thermophoresis ($N_t$) and Brownian motion ($N_b$) parameters. The impact of Schmidt number (Sc) on Sherwood number ($Sh_x^*$) is illustrated in Fig. 8e. The Sherwood number ($Sh_x^*$) increases with increment in both Schmidt number (Sc) and unsteadiness parameter (A). Figure 8f portrays the effect of chemical reaction parameter (${{\tilde{\sigma }}}_1$) on Sherwood number ($Sh_x^*$). There is a decrement in Sherwood number ($Sh_x^*$) with increasing values of chemical reaction parameter (${{\tilde{\sigma }}}_1$) since raising the chemical reaction rate increases the thickness of mass transfer boundary layer. Figure 8g,h illustrates the impact of thermophoresis ($N_t$) and Brownian motion ($N_b$) parameters on Sherwood number ($Sh_x^*$). There is slight increment in Sherwood number ($Sh_x^*$) values with increasing values of thermophoresis parameter ($N_t$) whereas a declination in Sherwood number ($Sh_x^*$) values is observed with Brownian motion parameter ($N_b$).

**Table 4:** For A = 0 and different values of $S_v, S_t, S_c$ with M = 3, Gr = 2, Pr = 7, Gc = 2, Ec = 0.5, and Sc = 0.6 the values of ${{\tilde{\chi }}}''(0),-{{\tilde{\zeta }}}'(0),-{{\tilde{\phi }}}'(0)$.

$S_{\nu }$	$S_{t}$	$S_{c}$	${\tilde{\chi }}''(0)$	$-{\tilde{\zeta }}'(0)$	$-{\tilde{\phi }}'(0)$
0.5	0.5	0.5	− 0.7542	0.6339	0.6484
1	0.5	0.5	− 0.4904	0.8020	0.6355
0.5	1	0.5	− 0.7615	0.3755	0.6477
0.5	0.5	1	− 0.8100	0.6908	0.4844

**Table 5:** For A = 0.5 and different values of $S_v, S_t, S_c$ with M = 3, Gr = 2, Pr = 7, Gc = 2, Ec = 0.5, and Sc = 0.6 the values of ${{\tilde{\chi }}}''(0),-{{\tilde{\zeta }}}'(0),-{{\tilde{\phi }}}'(0)$.

$S_{\nu }$	$S_{t}$	$S_{c}$	${\tilde{\chi }}''(0)$	$-{\tilde{\zeta }}'(0)$	$-{\tilde{\phi }}'(0)$
0.5	0.5	0.5	− 0.8237	0.8362	0.6977
1	0.5	0.5	− 0.5336	1.0014	0.6859
0.5	1	0.5	− 0.8327	0.4915	0.6970
0.5	0.5	1	− 0.8748	0.8753	0.5136

## Conclusions

The present study performs entropy generation minimization on mixed convective flow across a vertical stretching sheet with an inclined magnetic field, thermophoresis, Brownian motion, viscous dissipation, higher-order endothermic/exothermic chemical reaction with activation energy, and Joule heating. The RK-4 method, in combination with the shooting method, has been used to solve the resulting set of ODEs. The numerically obtained results have been compared to those published in the literature, and the results were found to be in good agreement. The following are some of the most important findings of the research:

- A declination in entropy profiles is observed with an increase in thermophoresis ($N_t$) and Brownian motion ($N_b$) parameters, while Bejan number profiles show an increment.
- An increment in inclination parameter ($\xi$) and velocity slip ($S_v$) exhibits declination in the velocity profiles.
- The dimensionless temperature profile declines with an enhancement in the values of Prandtl number (Pr), activation energy parameter (${\tilde{E}}^*$) and order of chemical reaction (N), whereas an increment is observed with Eckert number (Ec), thermophoresis ($N_t$) and Brownian motion ($N_b$) parameters.
- There is reduction in dimensionless concentration profiles with increasing values of Schmidt number (Sc), chemical reaction parameter (${{\tilde{\sigma }}}_1$) and concentration slip ($S_c$).
- Nusselt number ($Nu_x^*$) enhances with increment in Prandtl number (Pr), thermophoresis ($N_t$) and Brownian motion ($N_b$) parameters.
- A decrement in the skin-friction coefficient ($C_f^*$) is observed with increment in thermal slip ($S_t$) and concentration slip ($S_c$) whereas it increases with increment in velocity slip ($S_v$).
- Sherwood number ($Sh_x^*$) decreases with an increment in the values of thermal slip ($S_t$), concentration slip ($S_c$), and velocity slip ($S_v$), respectively.

Entropy generation minimization addressed in the present problem is helpful in several sectors of mainstream thermal engineering and science: cryogenics, heat transfer, education, storage systems, solar power plants, nuclear and fossil power plants, and refrigerators. Further, the applications for heat and mass transfer of the boundary layer flow across a stretching sheet are numerous and diverse, including manufacturing artificial film and fibers and some uses for diluted polymer solutions in the polymer processing sector. Multiple industries, including food processing, water mechanics, oil storage and geothermal energy production, base liquid mechanics, and oil emulsification, use the mass transfer process along with endothermic/exothermic chemical reactions, activation energy, and other related phenomena. The results of this problem can be used in various systems susceptible to considerable fluctuations in gravitational force, heat exchanger designs, wire and glass fiber drafting, and nuclear engineering concerning reactor cooling.

## Data Availability

The datasets used and/or analyzed during the current study available from the corresponding author on reasonable request.

## List of symbols

A: Unsteadiness parameter
${\tilde{B}}$: Uniform magnetic field
Br: Brinkmann number
${\tilde{C}}_f$: Concentration of the corresponding fluid
$C_f^*$: Coefficient of skin-friction
${\tilde{C}}_p^*$: Specific heat at constant pressure
${\tilde{C}}_w$: Concentration at the wall
$C_\infty ^*$: Ambient concentration of the fluid
${\tilde{D}}_b$: Brownian diffusion coefficient
${\tilde{D}}_t$: Thermophoretic diffusion coefficient
${\tilde{E}}$: Slip corresponding to velocity
${\tilde{E}}_a$: Dimensional activation energy
${\tilde{E}}^*$: Dimensionless activation energy
Ec: Eckert number
${\tilde{F}}$: Slip corresponding to temperature
${\tilde{g}}$: Acceleration by virtue of gravity
${\tilde{G}}$: Slip corresponding to concentration
Gr: Grashof number
Gc: Solutal Grashof number
$${\tilde{k}}^*$$: Thermal conductivity
$${\tilde{k}}_1$$: Boltzmann constant
m: Fitted rate constant
$${\tilde{m}}_w^*$$: Mass flux
M: Hartmann number
N: Order of chemical reaction
$N_b$: Thermophoretic motion parameter
$N_t$: Brownian motion parameter
$Nu_x^*$: Local Nusselt number
Pr: Prandtl number
${\tilde{q}}_w^*$: Heat flux at the surface
${\tilde{R}}$: Universal gas constant
Re: Reynolds number
S: Suction parameter
Sc: Schmidt number
$Sh_x^*$: Local Sherwood number
$S_c$: Dimensionless concentration slip
$S_t$: Dimensionless thermal slip
$S_v$: Dimensionless velocity slip
${\tilde{T}}_f$: Temperature of the corresponding fluid
${\tilde{T}}_w$: Temperature at the wall
$T_\infty ^*$: Ambient temperature of the fluid
${\tilde{u}}_1^*$: Velocity component in $x_1^*$-direction
${\tilde{U}}_w^*$: Stretching velocity of the sheet
${\tilde{v}}_1^*$: Velocity component in $y_1^*$-direction
${\tilde{V}}_w^*$: Velocity of suction or injection at the wall of the surface

## Greek letters

$\beta$: Coefficient of thermal expansion
$\beta ^*$: Coefficient of concentration expansion
$\beta _1^*$: Endothermic/exothermic coefficient
$${\tilde{\chi }}$$: Dimensionless velocity
$\delta ^*$: Temperature ratio parameter
$$\eta$$: Similarity variable
${\tilde{\lambda }}_1$: Endothermic/exothermic reaction variable
${\tilde{\mu }}^*$: Fluid’s dynamic viscosity
${\tilde{\nu }}^*$: Fluid’s kinematic viscosity
$\Omega$: Temperature difference parameter
$${\tilde{\phi }}$$: Dimensionless concentration
$$\psi ^*$$: Stream function
$\Psi$: Concentration difference parameter
$${\tilde{\rho }}^*$$: Fluid’s density
$${\tilde{\sigma }}$$: Electrical conductivity
${\tilde{\sigma }}_1$: Chemical reaction parameter
${\tilde{\tau }}^*$: Heat capacity ratio
${\tilde{\tau }}_w^*$: Shear stress at the wall
$\xi$: Inclination of magnetic field
$${\tilde{\zeta }}$$: Dimensionless temperature
$$\Lambda$$: Constant parameter
